# Multi-locus genotypes of *Enterocytozoon bieneusi* in captive Asiatic black bears in southwestern China: High genetic diversity, broad host range, and zoonotic potential

**DOI:** 10.1371/journal.pone.0171772

**Published:** 2017-02-09

**Authors:** Lei Deng, Wei Li, Zhijun Zhong, Chao Gong, Xuefeng Cao, Yuan Song, Wuyou Wang, Xiangming Huang, Xuehan Liu, Yanchun Hu, Hualin Fu, Min He, Ya Wang, Yue Zhang, Kongju Wu, Guangneng Peng

**Affiliations:** 1 The Key Laboratory of Animal Disease and Human Health of Sichuan Province, College of Veterinary Medicine, Sichuan Agricultural University, Chengdu, Sichuan Province, China; 2 Chengdu Giant Panda Breeding Research Base, Chengdu, Sichuan Province, China; National Cheng Kung University, TAIWAN

## Abstract

*Enterocytozoon bieneusi* is an obligate eukaryotic intracellular parasite that infects a wide variety of vertebrate and invertebrate hosts. Although considerable research has been conducted on this organism, relatively little information is available on the occurrence of *E*. *bieneusi* in captive Asiatic black bears. The present study was performed to determine the prevalence, genetic diversity, and zoonotic potential of *E*. *bieneusi* in captive Asiatic black bears in zoos in southwestern China. Fecal specimens from Asiatic black bears in four zoos, located in four different cities, were collected and analyzed for the prevalence of *E*. *bieneusi*. The average prevalence of *E*. *bieneusi* was 27.4% (29/106), with the highest prevalence in Guiyang Zoo (36.4%, 16/44). Altogether, five genotypes of *E*. *bieneusi* were identified among the 29 *E*. *bieneusi*-positive samples, including three known genotypes (CHB1, SC02, and horse2) and two novel genotypes named ABB1 and ABB2. Multi-locus sequence typing using three microsatellites (MS1, MS3, and MS7) and one minisatellite (MS4) revealed V, III, V, and IV genotypes at these four loci, respectively. Phylogenetic analysis showed that the genotypes SC02 and ABB2 were clustered into group 1 of zoonotic potential, the genotypes CHB1 and ABB1 were clustered into a new group, and the genotype horse2 was clustered into group 6 of unclear zoonotic potential. In conclusion, this study identified two novel *E*. *bieneusi* genotypes in captive Asiatic black bears, and used microsatellite and minisatellite markers to reveal *E*. *bieneusi* genetic diversity. Moreover, our findings show that genotypes SC02 (identified in humans) and ABB2 belong to group 1 with zoonotic potential, suggesting the risk of transmission of *E*. *bieneusi* from Asiatic black bears to humans and other animals.

## Introduction

Microsporidia, which are emerging obligate intracellular pathogens, have been classified as fungi. These organisms have been shown to infect invertebrate and vertebrate hosts worldwide [1]. To date, the phylum Microsporidia is composed of approximately 1,300 species in 160 genera, and at least 14 microsporidian species have been reported in humans [2, 3]. Among them, *Enterocytozoon bieneusi*, which is one of the most prevalent, is responsible for over 90% of cases of microsporidiosis in humans [4]. Individuals infected with *E*. *bieneusi* may shed spores into the environment via feces, resulting in an increased risk of transmission to other animals, including humans, through consumption of contaminated food and water [5]. In humans, clinical manifestations of microsporidiosis caused by *E*. *bieneusi* include life-threatening diarrhea and weight loss in individuals with deficient immune systems, such as those with AIDs, transplant recipients, and patients with cancer. In healthy individuals, *E*. *bieneusi* causes self-limiting diarrhea and malabsorption [6]. Following the first discovery of *E*. *bieneusi* infection in a Haitian patient with AIDS, several studies have been performed on the prevalence, genetic diversity, and route of transmission of this pathogen in various animals worldwide [2, 7].

The internal transcribed spacer (ITS) of the rRNA gene, which possesses considerable genetic variation, has been widely used for genotyping *E*. *bieneusi* isolates in humans and animals [6, 8]. To date, more than 240 genotypes of *E*. *bieneusi* have been identified based on intra-species DNA sequence variation in the ITS region [9–11]. Among these genotypes, 34 have been reported to occur only in humans, 11 have been recognized in humans and various animals, and other genotypes are host-specific [6]. The published ITS genotypes of *E*. *bieneusi* have been divided into nine different groups based on phylogenetic analyses [12]: the first group, which is the largest human-pathogenic cluster termed group 1, contains more than 94% of the published genotypes of *E*. *bieneusi* isolated from humans and animals. The remaining eight major clusters, named group 2 to group 9, are mostly found in specific hosts and wastewater [13]. High-resolution multi-locus sequence typing (MLST) using three microsatellites (MS1, MS3, and MS7) and one minisatellite (MS4) as markers has been developed [14] to elucidate the genetic diversity and the route of transmission of *E*. *bieneusi*.

In China, *E*. *bieneusi* has been identified in humans, livestock, pet animals, and captive wildlife; however, only limited studies have been conducted on the prevalence and genetic diversity of this pathogen in captive Asiatic black bears. Asiatic black bears are frequently found in Chinese zoos as commercial and ornamental animals, and are therefore in close contact with zoo-keepers. As a result of the high-density feeding environment in zoos and the potential for exposure to bear feces, infective spores of *E*. *bieneusi* from Asiatic black bears may pose a potential risk to other animals and public health. Therefore, the aims of the present study were to determine the prevalence of *E*. *bieneusi* in captive Asiatic black bears in zoos. In addition, we evaluated the genetic diversity of *E*. *bieneusi* by ITS sequencing and MLST analysis to assess implications for public health.

## Methods

### Ethics statement

The present study protocol was reviewed and approved by the Research Ethics Committee and the Animal Ethical Committee of Sichuan Agricultural University. Appropriate permission was obtained from zoo managers before the collection of fecal specimens.

### Specimen collection

In total, 106 fecal specimens were collected between October 2015 and September 2016 from captive Asiatic black bears in zoos in the Sichuan and Guizhou provinces of southwestern China (Table 1). Fresh fecal specimens from each Asiatic black bear were collected immediately after defecation on the ground and transferred into clean 50-ml plastic containers individually. All animals were healthy and none showed any clinical signs of gastrointestinal disease at the time of sampling. All fecal specimens were stored at 4°C in 2.5% (w/v) potassium dichromate.

**Table 1:** Prevalence and genotypes of *E*. *bieneusi* in Asiatic black bears from zoos in Southwestern China.

Locations (province)	Zoo	No. of examined	No. of positive (%)	Genotypes (n)
Sichuan	Chengdu	48	10 (20.8)	SC02 (6); CHB1 (3); ABB1 (1)
	Xichang	6	1 (16.7)	CHB1(1)
	Panzhihua	8	2 (25.0)	CHB1(2)
Guizhou	Guiyang	44	16 (36.4)	CHB1 (13); horse2 (2); ABB2 (1)
Total		106	29 (27.4)	CHB1 (19); SC02 (6); horse2 (2); ABB1 (1); ABB2 (1)

### DNA extraction

Fecal specimens were processed by sieving; then, samples were concentrated and washed three times with distilled water by centrifugation for 10 minutes at 1500 × *g*. Genomic DNA was extracted from approximately 200 mg of processed specimens, using an EZNA^®^ Stool DNA kit (Omega Biotek, Norcross, GA, USA) according to the manufacturer’s protocol. DNA was eluted in 200 μl of absolute ethanol and stored at -20°C until use for PCR analysis.

### PCR amplification

Extracted DNA was examined for the presence of *E*. *bieneusi* by nested PCR amplification of a 389-bp nucleotide fragment of the rRNA gene containing 76 bp of the 3’-end of SSU rRNA gene, 243 bp of the ITS region, and 70 bp of the 5’-region of LSU rRNA gene. Positive specimens were further characterized by MLST analyses using the MS1, MS3, MS4, and MS7 loci. The primers and cycling parameters employed for these reactions were as previously described (S1 Table) [14, 15]. TaKaRa Taq^™^ DNA Polymerase (TaKaRa Bio, Otsu, Japan) was used for all PCR amplifications. A negative control with no DNA added was included in all PCR tests. All secondary PCR products were subjected to electrophoresis on a 1% agarose gel containing ethidium bromide.

### Nucleotide sequencing and analysis

The secondary PCR products of anticipated size were directly sequenced by Life Technologies (Guangzhou, China), using a BigDye^®^ Terminator v3.1 cycle sequencing kit (Applied Biosystems, Carlsbad, CA, USA). Sequence accuracy was confirmed by two-directional sequencing and the sequencing of a new PCR product if necessary.

Nucleotide sequences obtained in the present study were aligned with each other and reference sequences downloaded from GenBank using the Basic Local Alignment Search Tool (BLAST) (http://www.ncbi.nlm.nih.gov/BLAST/) and ClustalX 1.83 (http://www.clustal.org/) to determine the genotypes of *E*. *bieneusi*. The genotypes were assigned previously published names if found to be identical to known genotypes. Genotypes with single nucleotide substitutions, deletions, or insertions in 243 bp of the ITS gene region of *E*. *bieneusi* relative to the known genotypes were considered novel genotypes, and named according to the established nomenclature system [16].

### Phylogenetic analysis

To assess the genetic relationships between the *E*. *bieneusi* genotypes in the present study and reference sequences previously published in GenBank, phylogenetic analysis was performed by constructing a neighboring-joining tree using Mega 6 software (http://www.megasoftware.net/), which is based on evolutionary distances calculated using a Kimura 2-parameter model. The reliability of these trees was assessed using bootstrap analysis with 1,000 replicates.

### Statistical analysis

The chi-squared test was performed to compare *E*. *bieneusi* infection rates between different sampling zoos, and differences were considered significant when P < 0.05.

### Nucleotide sequence accession numbers

Representative nucleotide sequences were deposited into the GenBank database under the following accession numbers: KY021392 to KY021396 for the rRNA gene ITS sequences of *E*. *bieneusi* (S2 Table), and KY021397 to KY021414 for the microsatellite loci (MS1, MS3, and MS7) and minisatellite (MS4).

## Results

### Prevalence of *E*. *bieneusi* in Asiatic black bears

In total, 29 of the 106 fecal specimens from Asiatic black bears (27.4%) were found to be positive for *E*. *bieneusi* (Table 1) via ITS-PCR amplification. All tested zoos showed evidence of *E*. *bieneusi* infection (Table 1), and the infection rate of *E*. *bieneusi* in different zoos was 16.7–36.4%. The highest infection rate of *E*. *bieneusi* was found to occur in Guiyang Zoo in the Guizhou province (36.4%, 16/44), followed by Panzhihua Zoo (25.0%, 2/8), Chengdu Zoo (20.8%, 10/48), and Xichang Zoo (16.7%, 1/6) in the Sichuan province; however, the differences in infection rate among the four zoos were not statistically significant (*χ*^*2*^ = 3.191, *df* = 3, P > 0.05).

### Genetic characterization and genotype distribution of *E*. *bieneusi* in Asiatic black bears

**Fig 1:**
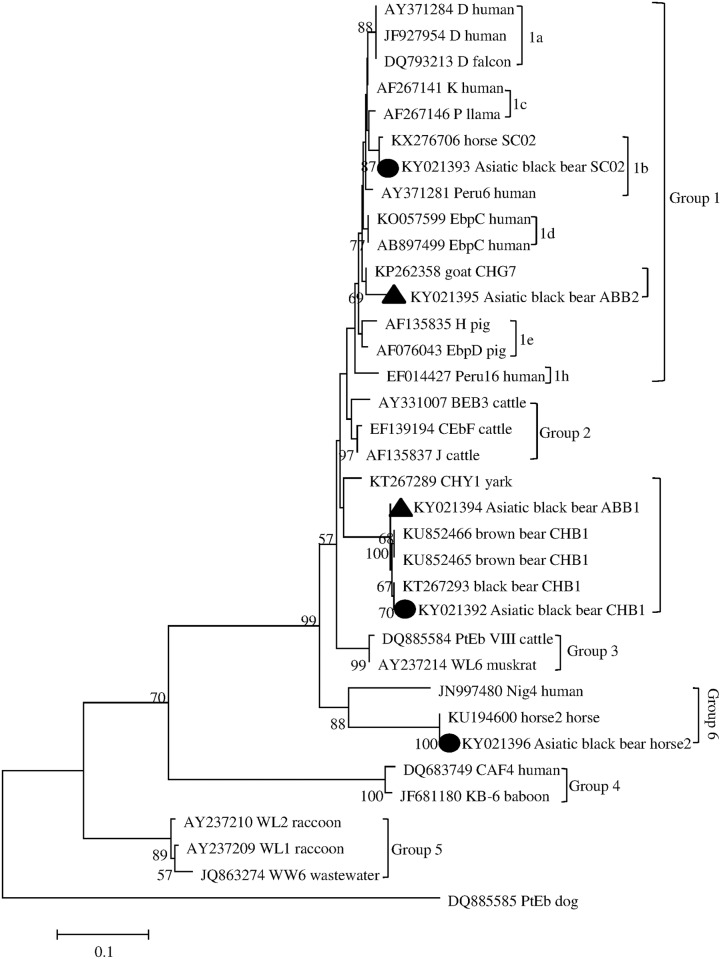
Phylogenetic relationship among *Enterocytozoon bieneusi* groups; the relationship between *E*. *bieneusi* genotypes identified in this study and other known genotypes deposited in GenBank was inferred by neighbor-joining analysis of ITS sequences based on genetic distance using the Kimura-2-parameter model. The numbers on the branches represent percent bootstrapping values from 1,000 replicates, with more than 50% shown in the tree. Each sequence is identified by its accession number, genotype designation, and host origin. Genotypes marked with *black triangles* and *black circles* are novel and known genotypes identified in this study, respectively.

Five genotypes were identified by sequence analysis of the ITS gene in 29 *E*. *bieneusi*-positive specimens (16 from Guizhou and 13 from Sichuan province), including three known genotypes (CHB1, SC02, and horse2) and two novel genotypes, which were named ABB1 and ABB2. Genotype CHB1, which was identified in the largest number of *E*. *bieneusi*-positive specimens (65.5%, 19/29), was the also most prevalent genotype in Guiyang Zoo, Panzhihua Zoo, and Xichang Zoo, followed by genotype SC02 (20.7%, 6/29), which was only detected in Chengdu Zoo. Genotype horse2 (6.9%, 2/29) was only identified in Guiyang Zoo.

Two and five single nucleotide polymorphisms (SNPs) within the 243-bp region of the ITS gene sequence of *E*. *bieneusi* were found in the two novel genotypes relative to the known genotypes. Genotype ABB1 had two SNPs (insertions: T and G) relative to genotype CHB1 (KU852466), with 99% homology. Genotype ABB2 had five SNP (substitutions: G/A, G/A, T/C, G/A, and A/G) relative to genotype CHG7 (KP262358).

### Phylogenetic analysis

Phylogenetic analysis by the neighbor-joining method based on the ITS gene sequences of *E*. *bieneusi* indicated that the five genotypes obtained in the present study belonged to three distinct groups: SC02 was clustered into group 1 and further classed into subgroup 1b. Genotype ABB2 was also clustered into group 1; however, further classification of this genotype formed a cluster distinct from any known to occur in subgroup 1. Genotypes CHB1 and ABB1 formed a new cluster between groups 2 and Group 3. Genotype horse2 was clustered into group 6 (Fig 1).

### Multi-locus sequence typing of *E*. *bieneusi*

In the present study, the 29 specimens positive for ITS were further characterized using one minisatellite (MS4) locus and three microsatellite (MS1, MS3, and MS7) loci. Nine, eight, nine, and eight samples were successfully amplified at the MS1, MS3, MS4, and MS7 loci, respectively; however, only six samples were simultaneously positive at all four loci. Sequence analysis identified V, III, IV, and V types in each locus, and new II, I, II, and I types in the four loci, respectively. Six distinct MLGs (MLG1-6) were observed by sequence analysis: four distinct MLGs were identified in genotype SC02, MLG1 was observed in genotype CHB1, and MLG3 was observed in genotype ABB1 (Table 2).

**Table 2:** Multi-locus sequence typing of *Enterocytozoon bieneusi* in Asiatic black bears in Southwestern China.

Zoo	ITS Genotype	Multilocus genotypes					MLGs
		MS1	MS3	MS4	MS7	GenBank accession Nos.	
Guiyang	CHB1	Type I*	Type I	Type I	Type I	KY021399, KY021403, KY021408, KY021410	MLG1
Chengdu	SC02	Type II	Type I	Type II*	Type II	KY021398, KY021403, KY021406, KY021413	MLG2
Chengdu	SC02	Type III	NA	Type III	Type III	KY021397, NA, KY021407, KY021411	–
Guiyang	CHB1	Type I*	Type II*	Type I	NA	KY021399, KY021404, KY021408, NA	–
Chengdu	ABB1	Type II	Type III	Type I	Type IV	KY021398, KY021405, KY021408, KY021412	MLG3
Chengdu	SC02	Type III	Type I	Type III	Type II	KY021397, KY021403, KY021407, KY021413	MLG4
Guiyang	CHB1	Type IV	Type I	Type IV*	NA	KY021401, KY021403, KY021409, NA	–
Chengdu	SC02	NA	NA	NA	Type I	NA, NA, NA, KY021410	–
Chengdu	SC02	Type V*	Type I	Type III	Type IV	KY021402, KY021403, KY021407, KY021412	MLG5
Chengdu	SC02	Type III	Type I	Type III	Type V*	KY021397, KY021403, KY021407, KY021414	MLG6

Note: NA = not amplified

* Novel genotypes

## Discussion

*E*. *bieneusi*, an important zoonotic parasite, has been the focus of numerous epidemiological studies in humans and animals worldwide. *E*. *bieneusi* has been identified in a number of captive and wild animal species [17–20]. Recent studies have reported the detection of *Cryptosporidium parvum* in black bears; however, little is known about the prevalence of *E*. *bieneusi* in these animals [21, 22]. In the present study, an overall infection rate of 27.4% (29/106) was observed in Asiatic black bears from zoos; The *E*. *bieneusi* infection rate observed in this study was higher than the previously reported rates of 15.8% and 27% reported for captive wildlife in Zhengzhou Zoo and Bifengxia Zoo in China, respectively [17, 19], but lower than the rates of 29.8% reported for nonhuman primates in zoos in China [23]. However, relative to the wild black bear, the infection rate was significantly lower than the rate of 40% (2/5) observed in black bears in New York City [24]. The observed differences in the infection rate of *E*. *bieneusi* among different zoos may be explained by variations in feeding density, geography, management system, sample size, and climate.

Three known genotypes (CHB1, SC02, and horse2) and two novel genotypes (ABB1 and ABB2) were identified in this study by analyzing ITS gene sequences of *E*. *bieneusi*. Genotype CHB1, which was originally detected in black bears, showed the highest prevalence among *E*. *bieneusi*-positive specimens (65.5%, 19/29). This is consistent with previous findings that genotype CHB1 infects black bears at a higher rate than other species in Bifengxia Zoo [17]. To date, genotype CHB1 has been found in Tibetan blue bears, ring-tailed lemurs, brown bears, red pandas, and Malayan sun bears in China (Table 3) [17, 19]. Genotype SC02, the second most prevalent genotype among the *E*. *bieneusi-*positive isolates (20.7%, 6/29), was identified in humans in a recent study. This genotype is reported to infect a variety of hosts such as Tibetan blue bears, sun bears, northern raccoons, horses, and squirrels [17, 18]. Genotype horse2 is frequently found in horses, and was also identified in Asiatic black bears in this study. These results indicate that the three known genotypes of *E*. *bieneusi* have a broad host range, and that captive Asiatic black bears may act as a potential source of *E*. *bieneusi* to infect other animals due to the high-density feeding condition in zoos.

**Table 3:** Host ranges and geographical distributions of *Enterocytozoon bieneusi* genotypes identified in Southwestern China.

Genotype	City^a^ (no. of positive specimens)	Host (location^b^)	Reference
CHB1	Guiyang (13); Chengdu (3); Panzhihua (2); Xichang (1)	Tibetan blue bear (Chengdu); Malayan sun bear (Chengdu); brown bear (Yaan); red panda (Yaan); ring-tailed lemur (Yaan); black bear (Zhengzhou)	[17, 19]
SC02	Chengdu (6)	Human (Yaan); Tibetan blue bear (Chengdu); sun bear (Chengdu); northern raccoon (Yaan); horse (Chengdu, Kunming); squirrel (Yaan)	[17, 18, 25]
horse2	Guiyang (2)	horse (Kunming); black bear	[25, 26]
ABB1	Chengdu (1)	black bear	This study
ABB2	Guiyang (1)	black bear	This study

^a^ City where *E*. *bieneusi* was identified in this study.

^b^ Location where *E*. *bieneusi* was identified in China prior to this work

A phylogenetic analysis based on a neighboring-joining tree of ITS gene sequences showed the genetic relationship of the five obtained genotypes of *E*. *bieneusi* with the known genotypes. The two genotypes SC02 and ABB2 belonged to group 1, which is composed of genotypes almost exclusively from humans, indicating their potential for zoonotic transmission and risks to public health [27–29]. Genotypes CHB1 and ABB1 were classified into a new group, which contained genotypes almost exclusively from bears, although a recent study demonstrated that some genotypes also belonged to a new group: genotypes CHY1 in Yak, CHK1 in white kangaroos, and CHK2 in gray kangaroos [19]. The genotype horse2 was clustered into group 6; this group was first identified in urban wastewater and also infects a broad range of hosts [30]. The genotypes gorilla 3 from gorillas, KB-5 and Macaque1 from non-human primates, YNH1 and YNH2 from horses, and Nig4 and Nig6 from humans have been classified into group 6 [25, 31–33]. Therefore, further molecular epidemiological studies are required to investigate the potential ability of the identified genotypes in the new group and in group 6 to cause microsporidiosis in humans and other animals.

It is not clear whether isolates of *E*. *bieneusi* with the same ITS gene sequences are genetically identical, or whether meiotic recombination occurs during the life cycle of this parasite [30, 34]. Therefore, the use of a single ITS gene marker may be inadequate for identifying all genotypes. Recently, a high-resolution MLST tool for subtyping *E*. *bieneusi* was developed [14]. High MLG diversity has been widely observed in the same ITS gene sequences in recent studies [17, 18, 23, 25, 35–37]. In the present study, nine, eight, nine, and eight samples were successfully amplified at the MS1, MS3, MS4, and MS7 loci, respectively. Six distinct MLGs (MLG1-6) were observed; these included four MLGs in genotype SC02 and one MLG each in genotypes CHB1 and ABB1 (Table 2). Our findings provide novel insights into the genetic diversity of *E*. *bieneusi* in Asiatic black bears.

In conclusion, this study revealed the prevalence (27.4%, 29/106) of zoonotic *E*. *bieneusi* in captive Asiatic black bears in Southwestern China. This study also found a broad host range for *E*. *bieneusi* genotypes CHB1, SC02, and horse2, and two novel genotypes (ABB1 and ABB2) in captive Asiatic black bears. Genetic diversity was observed using the ITS and MLST tool, and six distinct MLGs were identified in Asiatic black bears. The previous identification of genotype SC02 in humans indicates that Asiatic black bears may represent a potential host for transmission of microsporidiosis to humans and animals. Microsporidiosis may be transmitted between species, and no effective drugs are currently available to treat this disease. Our findings indicate the need for appropriate control strategies to prevent the transmission of this pathogen from captive Asiatic black bears to humans and other animals.

## Supporting information

S1 Table Genes, primers, sequence and annealing temperatures used in the PCRs and expected sizes of the PCR products. (DOCX)

S2 Table *E*. *bieneusi* ITS genotype, locations, host, and GenBank accession Nos. (DOCX)
