# Supplementary material for: Multi-locus genotypes of Enterocytozoon bieneusi in captive Asiatic black bears in southwestern China: High genetic diversity, broad host range, and zoonotic potential
Source: PLoS One. 2017 Feb 9;12(2):e0171772. doi: 10.1371/journal.pone.0171772 (PMC5300288; doi:10.1371/journal.pone.0171772)
Supplement: S1 Table — (DOCX) [file pone.0171772.s001.docx]

**S1 Table**

**Genes, primers, sequence and annealing temperatures used in the PCRs and expected sizes of the PCR products**

| **Gene locus** | **Primer** | **Sequence (5'-3')** | **Annealing temperature (°C)** | **Fragment length (bp)** | **References** |
| --- | --- | --- | --- | --- | --- |
| ITS | F1 | GATGGTCATAGGGATGAAGAGCTT | 55 | 410 | [15] |
|  | R1 | AATACAGGATCACTTGGATCCGT |  |  |  |
|  | F2 | AGGGATGAAGAGCTTCGGCTCTG | 55 | 392 |  |
|  | R2 | AATATCCCTAATACAGGATCACT |  |  |  |
| MS1 | F1 | CAAGTTGCAAGTTCAGTGTTTGAA | 58 | 843 | [14] |
|  | R1 | GATGAATATGCATCCATTGATGTT |  |  |  |
|  | F2 | TTGTAAATCGACCAAATGTGCTAT | 58 | 676 |  |
|  | R2 | GGACATAAACCACTAATTAATGTAAC |  |  |  |
| MS3 | F1 | CAAGCACTGTGGTTACTGTT | 55 | 702 | [14] |
|  | R1 | AGTTA GGGCATTTAATAAAATTA |  |  |  |
|  | F2 | GTTCAAGTAATTGATACCAGTCT | 55 | 537 |  |
|  | R2 | CTCATTGAATCTAAATGTGTATAA |  |  |  |
| MS4 | F1 | GCATATCGTCTCATAGGAACA | 55 | 965 | [14] |
|  | R1 | GTTCATGGTTATTAATTCCAGAA |  |  |  |
|  | F2 | CGA AGTGTACTACATGTCTCT | 55 | 885 |  |
|  | R2 | GGACTTTAATAAGTTACCTATAGT |  |  |  |
| MS7 | F1 | GTTGATCGTCCAGATGGAATT | 55 | 684 | [14] |
|  | R1 | GACTATCAGTATTACTGATTATAT |  |  |  |
|  | F2 | CAATAGTAAAGGAAGATGGTCA | 55 | 471 |  |
|  | R2 | CGTCGCTTTGTTTCATAATCTT |  |  |  |
