# Supplementary material for: Multi-locus genotypes of Enterocytozoon bieneusi in captive Asiatic black bears in southwestern China: High genetic diversity, broad host range, and zoonotic potential
Source: PLoS One. 2017 Feb 9;12(2):e0171772. doi: 10.1371/journal.pone.0171772 (PMC5300288; doi:10.1371/journal.pone.0171772)
Supplement: S2 Table — (DOCX) [file pone.0171772.s002.docx]

| **ITS Genotype** | **Locations (provinces)** | **Host** | **GenBank accession Nos.** |
| --- | --- | --- | --- |
| CHB1 | Sichuan, Guizhou | Asiatic black bear | KY021392 |
| SC02 | Sichuan | Asiatic black bear | KY021393 |
| horse2 | Guizhou | Asiatic black bear | KY021396 |
| ABB1 | Sichuan | Asiatic black bear | KY021394 |
| ABB2 | Guizhou | Asiatic black bear | KY021395 |

S2 Table ***E. bieneusi*** ITS genotype, locations, Host, and GenBank accession Nos.
